# Operando Evaluation of the Electrochemically Active Area in a Solid Oxide Fuel Cell Porous Electrode by Micro X‑ray Absorption Spectroscopy

**DOI:** 10.1021/acs.jpclett.5c02422

**Published:** 2025-09-06

**Authors:** Yoshinobu Fujimaki, Takashi Nakamura, Yuta Kimura, Kiyofumi Nitta, Oki Sekizawa, Yasuko Terada, Keiji Yashiro, Tatsuya Kawada, Koji Amezawa

**Affiliations:** † Institute of Multidisciplinary Research for Advanced Materials (IMRAM), 13101 Tohoku University, 2-1-1 Katahira, Aoba-ku, Sendai 980-8577, Japan; ‡ Japan Synchrotron Radiation Research Institute (JASRI), 1-1-1 Kouto, Sayo-cho, Sayo-gun, Hyogo 679-5198, Japan; § Graduate School of Environmental Studies, Tohoku University, 6-6-01 Aoba, Aramaki, Aoba-ku, Sendai 980-8579, Japan

## Abstract

An operando X-ray absorption spectroscopic technique, which enables us to measure X-ray absorption spectra with a position resolution of submicrometers at increased temperatures while controlling atmospheres and passing an electrical current through the specimen, was developed. By applying this technique, the electrochemically active area in a porous La_0.6_Sr_0.4_CoO_3−δ_ electrode for a solid oxide fuel cell (SOFC) was experimentally and directly evaluated for the first time. The characteristic length of the active area was approximately 1 μm from the electrode–electrolyte interface under a cathodic overpotential of 140 mV at 873 K under 10^–2^ bar of *P*(O_2_), although the investigated electrode was thicker than 50 μm. This demonstrated that a reaction in an SOFC electrode inhomogeneously proceeds in a very limited area near the electrode–electrolyte interface, even when a mixed ionic and electronic conducting oxide is used as the electrode, and that direct evaluation of the active area can provide guidelines for high-performance electrode design.

## Graphical abstract

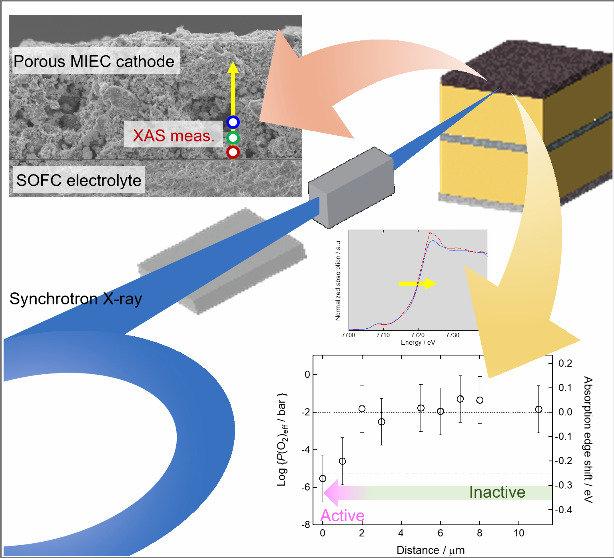

The solid oxide fuel cell (SOFC) is one of the promising candidates for next-generation environmentally friendly power sources for a sustainable society because of its high efficiency and fuel flexibility.^1–4^ SOFCs are typically operated around 973–1273 K, and such high-temperature operation causes several practical issues in terms of long-term reliability and durability. Decreasing the operating temperature is one solution for these issues, and hence, intermediate-temperature SOFCs have recently attracted much attention. However, decreasing the operating temperature results in serious performance degradation, especially in the cathode. In order to establish high-performance cathodes working at intermediate temperatures, we must understand the factors that govern the electrode reaction.

Mixed ionic and electronic conductors (MIECs), for instance, (La,Sr)­(Co,Fe)­O_3−δ_, are currently utilized as SOFC cathode materials.^2,3^ In an MIEC cathode, the electrochemical reaction can proceed not only at the triple-phase boundaries (gas–electrode–electrolyte) but also at the double-phase boundaries (gas–electrode). Possible elemental reaction steps in the MIEC cathode are considered as follows: (i) oxygen adsorption and dissociation on the electrode surface, (ii) ionization of the oxygen atom and incorporation into the electrode bulk, (iii) oxide ion diffusion in the electrode bulk, and (iv) oxide ion transfer from the electrode bulk into the electrolyte bulk. The rates of steps i and ii are described by surface exchange coefficient *k*, and that of the step iii is described by diffusion coefficient *D*. Since the resistance for oxide ion diffusion in an MIEC SOFC electrode increases with the distance from the electrode–electrolyte interface, the electrochemical reaction does not proceed homogeneously in the whole area of the practical porous electrode. The reaction current is considered to decrease with an increase in the distance from the interface. Therefore, to improve the electrode performance, it is important to understand how the reaction is distributed in the electrode and to optimize the electrode materials and microstructures.

Adler et al. theoretically discussed such an inhomogeneous reaction in a porous MIEC SOFC electrode.^5^ Based on their model, the so-called Adler–Lane–Steele (ALS) model, they simulated the profiles of the reaction current in a porous MIEC SOFC electrode under polarization and showed that the reaction distribution can be principally determined by the competition between the resistances for the surface reaction and oxide ion diffusion.^5,6^ The electrochemically active area is predicted to increase when the resistance is larger for the surface reaction or smaller for oxide ion diffusion. The resistances for the surface reaction and oxide ion diffusion can be determined by surface exchange coefficient *k* and diffusion coefficient *D* of the electrode material, respectively, if the electrode microstructures, such as tortuosity, particle size, surface area, and porosity, are known. Based on this idea, the electrochemically active area has been evaluated from *k* and *D* values.^5–15^ For instance, Lu et al. calculated the electrochemically active area in porous La_0.6_Sr_0.4_CoO_3−δ_ by using *k* and *D* values obtained from electrochemical impedance spectroscopy (EIS) measurement.^5^ By combining 3D reconstruction of the electrode microstructures and calculation by the finite element method (FEM) model while using *k* and *D* values from the literature, Carraro et al. have presented the electrochemically active area in a porous La_0.58_Sr_0.4_Co_0.2_Fe_0.8_O_3−δ_ electrode.^8^ Recently, attempts to predict the performance and the reaction distribution of SOFC electrodes were made using machine learning of the electrode microstructures, although the target was not MIEC but cermet fuel electrodes.^16^ There is no doubt that these evaluations are well-conceived for the examination of the distribution of the electrochemical reaction in SOFC electrodes. However, it is true that these are not direct observations, and no one could ensure the validity of the evaluations.

Considering the background mentioned above, in this work, we aimed to establish a methodology for the direct evaluation of the reaction distribution in an MIEC SOFC electrode near the infterce with an electrolyte. For this purpose, we developed operando high-temperature electrochemical micro X-ray absorption spectroscopy (XAS). This technique enables us to measure X-ray absorption spectra with a positional resolution higher than 1 μm, while controlling temperature and atmospheric conditions, and passing an electrical current through the electrode. Then, this operando local analytical technique was applied to investigate the reaction distribution in a porous La_0.6_Sr_0.4_CoO_3−δ_ electrode on a Ce_0.9_Gd_0.1_O_1.95_ electrolyte, which are a typical MIEC cathode and oxide ion-conducting electrolyte for SOFC, respectively. To the best of our knowledge, this is the first report that directly evaluated the reaction distribution in a practical porous SOFC electrode in an experimental manner.

X-ray absorption spectroscopy can give us information about the electronic and chemical states of the material investigated. For instance, in La_0.6_Sr_0.4_CoO_3−δ_ investigated in this study, it was reported that the mean oxidation state of the Co ion can be estimated from the shift of the absorption-edge energy in the Co K-edge XAS spectrum.^17,18^ According to our previous operando XAS measurements using a dense thin film of La_0.6_Sr_0.4_CoO_3−δ_ as the model electrode, we demonstrated that cathodic polarization partially reduces the electrode oxide even when the ambient *P*(O_2_) is constant.^19^ It was also shown that the electrode is reduced more as a larger electrode overpotential is applied, i.e., a larger reaction current is passed.^19^ Such “electrochemical reduction” suggested that the oxygen chemical potential is effectively decreased at the electrode surface due to cathodic polarization, because the surface reaction is the rate-determining elementary step in the case of electrochemical oxygen reduction on La_0.6_Sr_0.4_CoO_3−δ_.^20,21^ These findings tell us that the oxygen chemical potential changes depending on the amount of reaction current and therefore that the reaction distribution in the electrode can be directly clarified if the distribution of oxygen chemical potential is detected. In this study, by applying the developed operando high-temperature electrochemical micro XAS technique, the oxygen chemical potential at the desired position in a porous La_0.6_Sr_0.4_CoO_3−δ_ electrode was evaluated by detecting the change in the mean oxidation state of the Co ion. By repeating this measurement under constant cathodic polarization while changing the measuring point, we clarified the reaction distribution in the practical porous electrode as a function of the distance from the electrode–electrolyte interface.

In order to directly evaluate the reaction distribution in an MIEC SOFC electrode, an analysis technique that can analyze the electronic and chemical states of the electrode with sufficiently high positional resolution is required. In addition, the analysis should be carried out under SOFC operating conditions, namely, at increased temperatures in a controlled atmosphere while passing an electrical current through a specimen. With such an analytical technique, operando high-temperature electrochemical micro XAS was developed in this study. For XAS measurements with high positional resolution, the incident X-ray beam was focused into a 0.5 μm × 0.8 μm area by using a Kirkpatrick-Baez mirror. A three-terminal electrochemical cell was prepared and mounted atop an electric furnace in the sample holder for operando XAS measurements.^18,22^ Pictures of the experimental setup at the synchrotron beamline and the electrochemical cell on the sample holder are shown in Figure 1. The cross section of the cell was placed face up, as shown in the bottom left picture and bottom right schematic illustration of Figure 1. The focused X-ray was incident perpendicular to the cross section of the cell, so that the electronic and chemical states of the electrode material can be analyzed in the electrode thickness direction as a function of the distance from the electrode–electrolyte interface. XAS measurements were carried out in fluorescence mode. Since the attenuation length of the X-ray with the energy around the Co K-edge is calculated as 5 μm in La_0.6_Sr0._4_CoO_3−δ_, the observed signal of the fluorescence X-ray is thought to reflect the average electronic structure of the electrode within this length from the observation surface (the electrode cross section) in the direction of the incident X-ray. The measuring positions were determined based on the result of 2D elementary mapping by fluorescence X-ray analysis of Co Kα (electrode) and Ce Lα (electrolyte). The sample holder was covered by an Al-coated Kapton film, and the atmosphere inside the holder was kept constant by a gas flow. By using these setups, an X-ray absorption spectrum could be measured with a positional resolution higher than 1 μm at the desired position under controlled temperature, atmosphere, and polarization.

**Figure 1:**
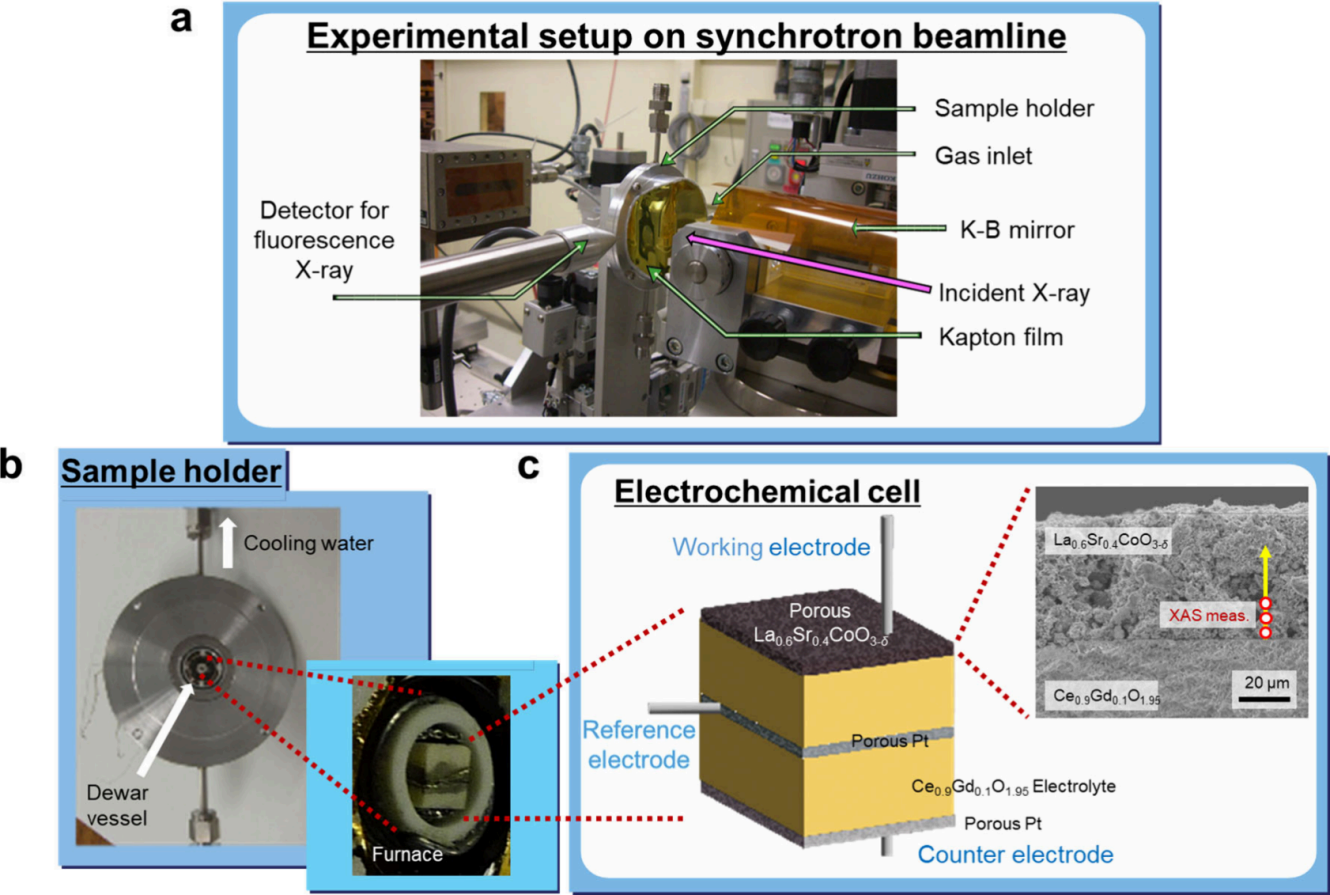
Experimental setup for operando high-tempearature electrochemical micro X-ray absorption spectroscopy measurements. (a) Overview of experimental setup on the synchrotron beamline. (b) Three-terminal electrochemical cells mounted on a sample holder. (c) Schematic illustration of the electrochemical cell and cross-sectional SEM image of the porous La_0.6_Sr_0.4_CoO_3−δ_ electrode investigated.

As mentioned in the beginning of this section, it is known that the Co K-edge absorption energy of La_0.6_Sr_0.4_CoO_3−δ_ is shifted depending on the oxygen chemical potential (oxygen partial pressure) to which the material is exposed. This happens because the increase or decrease in oxygen chemical potential causes a decrease or increase, respectively, in oxygen vacancy concentration δ and consequently a decrease or increase, respectively, in the mean valence of the Co ion in La_0.6_Sr_0.4_CoO_3−δ_. This indicates that the change in the oxygen chemical potential in La_0.6_Sr_0.4_CoO_3−δ_ can be evaluated by measuring the Co K-edge X-ray absorption spectra if the temperature is kept constant. In this work, for the purpose of evaluating the change in the oxygen chemical potential or effective oxygen partial pressure *P*(O_2_)_eff_, due to polarization, the Co K-edge X-ray absorption spectra of the porous La_0.6_Sr_0.4_CoO_3−δ_ electrode were first measured at various ambient *P*(O_2_) values under open circuit conditions at a constant temperature of 873 K, and then the calibration line, correlating the Co K-edge absorption energy with *P*(O_2_)_eff_, was obtained. In this series of measurements, the measuring position was fixed in the middle of the porous electrode.

Figure 2a shows the obtained Co K-edge X-ray absorption near-edge structure (XANES) spectra of the porous La_0.6_Sr_0.4_CoO_3−δ_ electrode under 1–10^–3^ bar of *P*(O_2_) under open circuit conditions at 873 K. The inset of Figure 2a shows a magnified view of the spectra near the absorption edge. The absorption energy was slightly decreased with ambient *P*(O_2_). A similar trend was found by ex situ measurements of the quenched samples in our previous papers.^17,18^ Figure 2b shows the shifts of the Co K-edge absorption energy as a function of ambient *P*(O_2_). Here, the absorption-edge energy was defined as the energy at which half of the absorbance change of the XANES spectrum was observed and that under 10^–2^ bar of *P*(O_2_) was taken as the reference state. As shown in Figure 2b, the absorption energy seemed to linearly decrease with ambient *P*(O_2_). The amount of energy shift was approximately 0.22 eV between 1 and 10^–3^ bar of *P*(O_2_) at 873 K. An approximately straight line calculated by the least-squares method is shown as a solid line in Figure 2b. By using this line as a calibration line, the change in *P*(O_2_)_eff_ due to polarization can be quantitatively estimated from Co K-edge XAS measurements.

**Figure 2:**
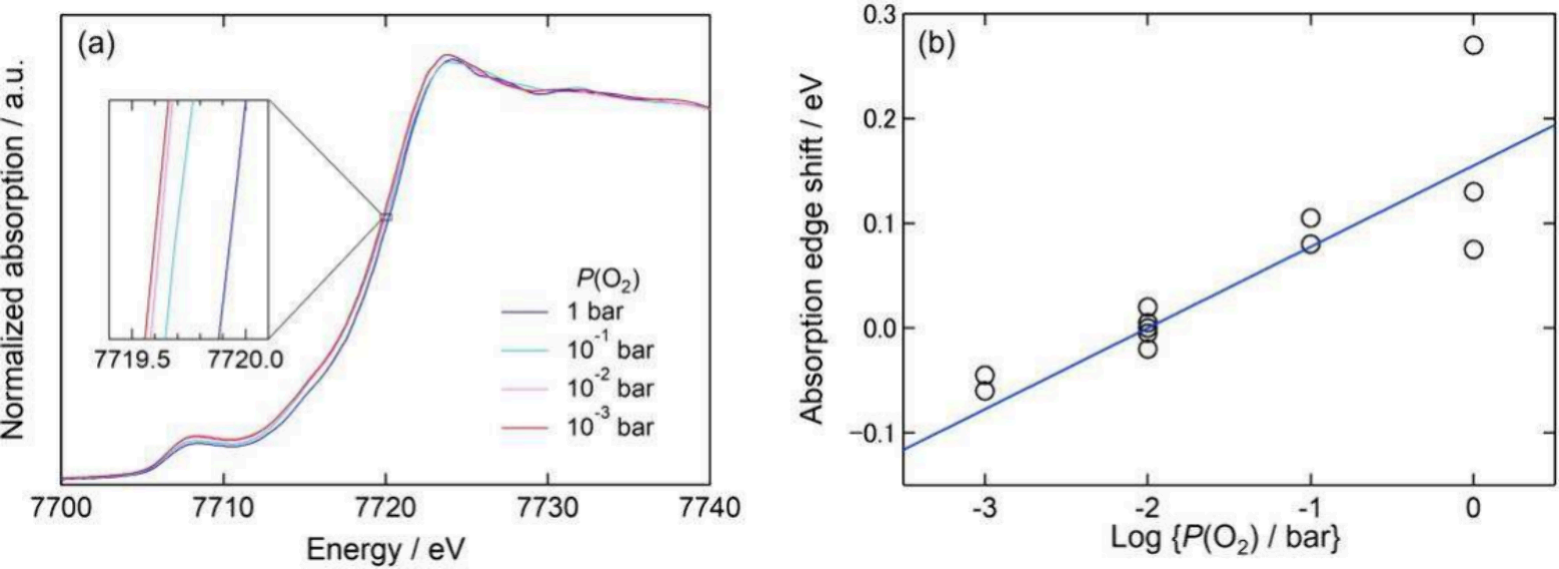
Co K-edge X-ray absorption spectroscopy measurements of La_0.6_Sr_0.4_CoO_3−δ_ at 873 K. (a) XANES spectra at various ambient oxygen partial pressures *P*(O_2_) under an open circuit. (b) Shift of the absorption-edge energy as a function of *P*(O_2_).

For the direct evaluation of the electrochemically active area, operando high-temperature electrochemical micro XAS measurements were carried out at the desired micro area in the porous La_0.6_Sr_0.4_CoO_3−δ_ electrode under cathodic polarization. Figure 3a shows the Co K-edge XANES spectra observed at various measuring positions under a constant cathodic overpotential of 140 mV and 873 K under 10^–2^ bar of *P*(O_2_). The experimental conditions were determined based on the practical operating conditions of the SOFC cathode and the ease of detecting the electrochemically active area by operando XAS measurement. In this figure, the spectrum observed under open circuit conditions is shown as the black curve for comparison. The position of the electrode–electrolyte interface was defined as a reference point, i.e., *x* = 0 μm. As shown in Figure 3a, the Co K-edge absorption energy was decreased at the positions near the electrode–electrolyte interface in spite of the constant ambient *P*(O_2_). The shift was gradually relaxed with an increase in the distance from the interface. At the positions far from the interface, the absorption energy was almost equivalent to that observed under open circuit conditions. These results suggested that, under cathodic polarization, (i) *P*(O_2_)_eff_ decreased near the electrode–electrolyte interface in the porous electrode even though the ambient *P*(O_2_) was kept constant and (ii) such a reducing condition due to cathodic polarization gradually relaxed with the distance from the electrode–electrolyte interface.

**Figure 3:**
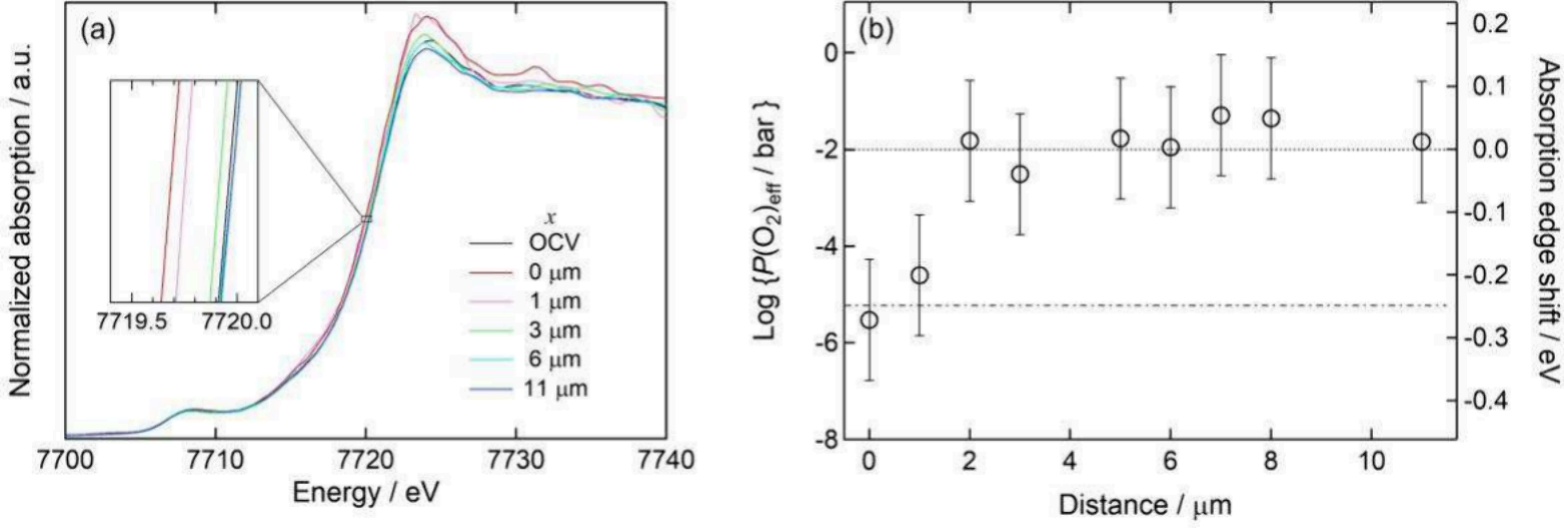
Operando Co K-edge X-ray absorption spectroscopy measurements of the porous La_0.6_Sr_0.4_CoO_3−δ_ electrode at 873 K, a *P*(O_2_) of 10^–2^ bar, and a cathodic overpotential of 140 mV. (a) Co K-edge XANES spectra observed at various positions in the electrode. (b) Distibution of effective oxygen partial pressure *P*(O_2_)_eff_ as a function of the distance from the electrode–electrolyte interface.

The *P*(O_2_)_eff_ under cathodic polarization at each measuring position was evaluated from the shift of the absorption-edge energy in the observed XANES spectra by using the calibration line in Figure 2b. Figure 3b gives *P*(O_2_)_eff_ as a function of the distance from the electrode–electrolyte interface. The dashed line in Figure 3b expresses the atmospheric *P*(O_2_), i.e., 10^–2^ bar. On the other hand, the dashed–dotted line gives the effective oxygen partial pressure at the electrode–electrolyte interface, *P*(O_2_)_eff,int_, which is expected from the applied cathodic overpotential. The relation between *P*(O_2_)_eff,int_ and applied overpotential η is described by the equation^20^

$$P{\left(O_{2}\right)}_{\mathrm{eff},\mathrm{int}}=P\left(O_{2}\right)\;\exp\left(\frac{4F\eta }{RT}\right)$$ 1

where *P*(O_2_) is the ambient oxygen partial pressure and *F*, *R*, and *T* are Faraday’s constant, the gas constant, and the absolute temperature, respectively. As shown in Figure 3b, *P*(O_2_)_eff_ drastically decreased in the vicinity of the electrode–electrolyte interface. *P*(O_2_)_eff_ at the interface evaluated from the operando micro XAS measurement was comparable to *P*(O_2_)_eff,int_ predicted from eq 1. *P*(O_2_)_eff_ gradually increased with the distance from the interface. In the area approximately 2 μm from the interface, *P*(O_2_)_eff_ became comparable to ambient *P*(O_2_).

As previously mentioned, under cathodic polarization, a decrease in the oxygen chemical potential is expected in the area where the electrode reaction takes place. The size of the decrease in the oxygen chemical potential, which corresponds to the decrease in log­{(*P*(O_2_)_eff_)}, reflects the amount of reaction current. Therefore, the result in Figure 3b demonstrates that the electrochemical reaction proceeded only within approximately 2 μm of the electrode–electrolyte interface in the porous La_0.6_Sr_0.4_CoO_3−δ_ electrode under a cathodic overpotential of 140 mV at 873 K in 10^–2^ bar of *P*(O_2_), although the electrode thickness was about 50 μm. In other words, only a very limited area in the electrode functions as the reaction sites even in a mixed ionic and electronic conducting electrode. This indicated that the thickness of the SOFC cathode can be made thinner than that in conventional SOFCs, although decreasing the electrode thickness may increase the resistance of the current collection in the in-plane direction of the electrode. To the best of our knowledge, this is the first study to directly and quantitatively observe the electrochemically active area in a practical SOFC porous electrode in an experimental manner. It is here noted that the length of the electrochemically active area possibly depends on the operating conditions (the magnitude of the overpotential, ambient *P*(O_2_), temperature, etc.) as well as electrode microstructures (porosity, specific surface area, tortuosity, etc.).

Adler et al. introduced the characteristic length, *l*_δ_, that characterizes the distance of the electrochemically active area from the electrode–electrolyte interface in a porous MIEC electrode.^5–8^ *l*_δ_ is defined as the distance between the electrode–electrolyte interface and the position where the amount of ionic current reaches 1/*e* of its maximum value at the interface. Considering Ohm’s law, the overpotential, or the oxygen chemical potential, at distance *l*_δ_ is also 1/*e* of that at the interface. Following this definition, the characteristic length of electrochemically active area *l*_δ_ in the porous La_0.6_Sr_0.4_CoO_3−δ_ electrode in this study was 1 μm or less.

The electrochemically active area in MIEC SOFC electrodes has been extensively investigated by electrochemical measurements and numerical calculations. These are indirect but practically convenient ways to estimate the electrochemically active area. Figure 4a presents a typical AC EIS of the porous La_0.6_Sr_0.4_CoO_3−δ_ electrode in this work, which was taken under an open circuit at 873 K in 10^–2^ bar of *P*(O_2_). A Gerischer-type impedance response was observed. The electrode reaction in an MIEC SOFC porous electrode is often described by a semi-infinite transmission line model, as shown in Figure 4b. For this configuration, impedance *Z*(ω) is given by^23,24^

$$Z\left(\omega \right)=\sqrt{\frac{R_{i}}{\frac{1}{R_{F}}+j\omega C}}$$ 2

where *R*_F_ and *C* are the resistance and the capacitance attributed to the electrode reaction, respectively, and *R*_i_ is the resistance of ionic transport in the electrode bulk. Then the reaction distribution, i.e., the Faradaic current as a function of the distance from the electrode–electrolyte interface, *I*(*x*), is expressed by

$$I\left(x\right)=I\left(0\right)\;\exp\left[-\sqrt{\left(\frac{1}{R_{F}}+j\omega C\right)R_{i}}x\right]$$ 3

Since *l*_δ_ is the distance between the electrode–electrolyte interface and the position where the amount of Faradaic current is *I*(0)/*e* under DC polarization (ω = 0), *l*_δ_ is described by *R*_F_ and *R*_i_

$$l_{\delta }=\sqrt{\frac{R_{F}}{R_{i}}}$$ 4

**Figure 4:**
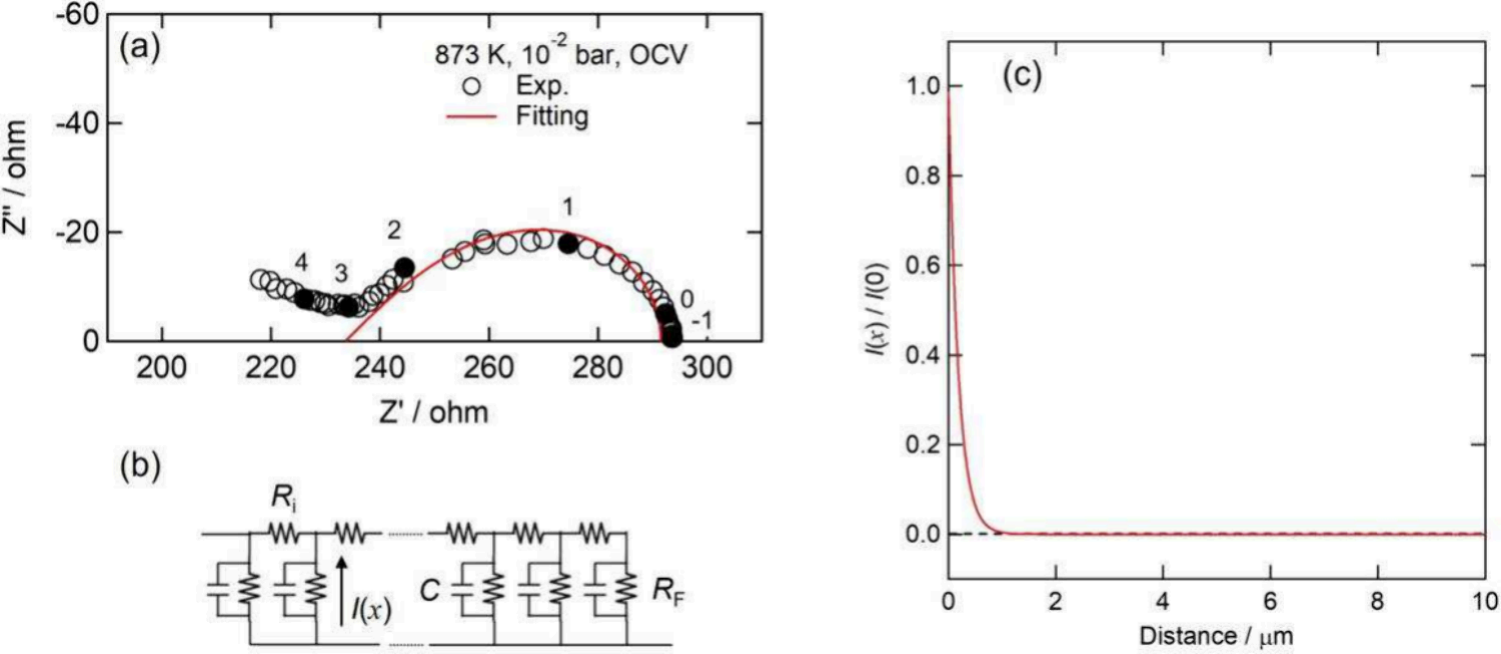
AC electrochemical impedance measurements of the porous La_0.6_Sr_0.4_CoO_3−δ_ electrode at 873 K and a *P*(O_2_) of 10^–2^ bar under open circuit conditions. (a) Nyquist plot and fitting curve by a semifinite transmission line model. The numbers in the figure represent the order of frequency. (b) Semifinite transmission line model used for the fitting. (c) Calculated distibution of the normalized Faradaic current as a function of the distance from the electrode–electrolyte interface.

Considering eqs 2–4, the reaction distribution can be estimated if the values of *R*_F_ and *R*_i_ are obtained from the fitting of the impedance spectrum by the transmission line model. The solid curve in Figure 4a presents the fitting result when assuming the transmission line model, and Figure 4c shows the reaction distribution estimated from the obtained *R*_F_ and *R*_i_. This distribution estimated from EIS is slightly precipitous compared with one directly observed by operando micro XAS, but it is reasonably similar in terms of the order of magnitude. Here it should be noted that *l*_δ_ values obtained from micro XAS and EIS measurements are different spatial information. *l*_δ_ obtained from micro XAS measurements is the local *l*_δ_ at the observed position. On the other hand, *l*_δ_ obtained from EIS measurements is the average *l*_δ_ across the entire electrode. Since microstructures of the porous electrode are complicated and not uniform depending on position, slightly different values of *l*_δ_ were thought to be obtained from micro XAS and EIS measurements.

Lu et al. reported a similar estimation of the electrochemically active area from EIS measurements for a porous La_0.6_Sr_0.4_CoO_3−δ_ electrode.^9^ They estimated *l*_δ_ as approximately 1 μm at 923 K under 10^–2^ bar of *P*(O_2_). This value is close to that in this work, confirming the validity of our results, although we cannot simply compare the results because the measurement conditions and the electrode microstructures are slightly different.

According to the theoretical treatment by Adler et al.,^5–9^ i.e., the ALS model, in the case of a mixed-conducting oxide like La_0.6_Sr_0.4_CoO_3−δ_, resistances *R*_F_ and *R*_i_ can be related to surface exchange coefficient *k* and diffusion coefficient *D* of the electrode material, respectively, if the electrode microstructures, such as porosity, specific surface area, and tortuosity, are known. On the other hand, capacitance *C* can be related to the thermodynamic factor for the oxygen nonstoichiometric change of the electrode material. As a result, it was shown that the electrochemically active area can be determined by the ratio between the diffusion coefficient and the surface exchange coefficient (*D*/*k*) when the electrode material and microstructures are the same. Based on this treatment, Carraro et al. numerically discussed the reaction distribution while including the real 3D electrode microstructures constructed by FIB-SEM analysis in the calculation.^12,13^ They simulated the dimensionless reaction current profile in a porous La_0.58_Sr_0.4_Co_0.2_Fe_0.8_O_3−δ_ electrode by using literature values of *k* and *D* and then obtained *l*_δ_ within 1 μm at 873 K in air. A similar three-dimensional numerical analysis was also performed by Matsuzaki et al., and *l*_δ_ in a porous La_0.6_Sr_0.4_Co_0.2_Fe_0.8_O_3−δ_ electrode was reported as 5 μm at 973 K in 0.20 bar of *P*(O_2_).^15^ Again, we cannot simply compare these results with ours, because the electrode materials, electrode microstructures, and experimental conditions were different. However, if *l*_δ_ is proportional to the square root of *D*/*k* as Adler et al. suggested,^5–9^ *l*_δ_ is presumed to increase with a decrease in *P*(O_2_) while insignificantly depending on overpotential and temperature. Assuming such changes in *l*_δ_ depending on the experimental conditions, the values of *l*_δ_ reported above reasonably agree with our results from XAS measurements in this study. These facts verify the theory that the electrochemically active area can be principally determined by *k* and *D*.

As discussed throughout this paper, we succeeded in establishing an experimental technique for the direct evaluation of the reaction distribution in an MIEC SOFC electrode. However, as shown in Figure 3b, the experimental error in *P*(O_2_)_eff_ estimated from XANES spectra is relatively large. Such a large experimental error is considered to arise mainly from the inhomogeneity of the electrode microstructure. Microstructures of a porous electrode are complicated and not uniform from position to position. Thus, the reaction distribution cannot be expressed simply only as a function of the distance from the electrode–electrolyte interface in the case of a porous electrode.^14,15^ On the other hand, the operando micro XAS technique developed in this work gives us the average *P*(O_2_)_eff_ in the direction of the incident X-ray, although it has a high position resolution in the in-plane direction perpendicular to the X-ray incident direction. To clarify the influence of the electrode microstructures on the reaction distribution, it would be effective to perform operando micro XAS measurements and 3D microstructural analysis with the same electrode and to compare the reaction distribution directly observed by XAS and that calculated by the ALS model while taking the electrode microstructures into account. To quantitatively investigate the influence of the electrode material on the reaction distribution, it would be useful to perform operando micro XAS measurements using model electrodes in which the electrode microstructure and geometry are well-defined. Both of these investigations are now in progress in our group, and we will report the results elsewhere in the near future.

The direct evaluation of the reaction distribution and electrochemically active area in a porous La_0.6_Sr_0.4_CoO_3−δ_ electrode near the interface with a Ce_0.9_Gd_0.1_O_1.95_ electrolyte was successfully accomplished by using operando high-temperature electrochemical micro XAS measurements. The characteristic length of the electrochemically active area was evaluated as approximately 1 μm from the electrode–electrolyte interface under a constant cathodic overpotential of 140 mV at 873 K and 10^–2^ bar of *P*(O_2_). The results obtained in this work demonstrated that the electrochemical reaction proceeds inhomogeneously in a porous SOFC electrode, and only part of the electrode within several micrometers of the electrode–electrolyte interface serves as the reaction sites even when a mixed ionic and electronic conductor is used as an electrode material. Such information about the reaction distribution is essential for the design and optimization of the material and microstructure for high-performance MIEC SOFC electrodes. Moreover, the methodology for interfacial analysis developed in this work can be a general tool, which is applicable to investigate the inhomogeneous reaction and its distribution not only in SOFC but also for various devices, such as other types of fuel cells, electrolysis cells, and even batteries, including all-solid-state lithium ion batteries.

## Experimental Section

*Sample Preparation*. A three-terminal electrochemical cell was fabricated with a dense Ce_0.9_Gd_0.1_O_1.95_ electrolyte cube. A schematic illustration of the prepared cell is presented in Figure 1. La_0.6_Sr_0.4_CoO_3−δ_ powder was screen-printed on the sintered compact of Ce_0.9_Gd_0.1_O_1.95_ with an organic solvent (TMS-1, Tanaka Holdings Co., Ltd.) and fired at 1273 K for 6 h. Detailed procedures for the synthesis of Ce_0.9_Gd_0.1_O_1.95_ and La_0.6_Sr_0.4_CoO_3−δ_ are provided in the Supporting Information. The obtained Ce_0.9_Gd_0.1_O_1.95_ and La_0.6_Sr_0.4_CoO_3−δ_ powders were confirmed as single-phase fluorite and perovskite, respectively (see Figures S1 and S2). The thickness of the prepared cathode was about 50 μm, as shown in the cross-sectional SEM image in Figure 1. From the SEM observation, it was estimated that the particle size and porosity of the electrode were approximately 0.5 μm and 0.5, respectively. A porous Pt (TR-7907, Tanaka Holdings Co., Ltd.) was put on the back and lateral sides of the electrolyte cube as a counter electrode and a reference electrode, respectively.

*Operando XAS Measurement*. Operando micro XAS measurements were performed at beamline BL37XU, SPring-8, Japan Synchrotron Radiation Research Institute (JASRI). In this study, the incident X-ray beam from the synchrotron radiation was focused onto a 0.5 μm × 0.8 μm area using a Kirkpatrick-Baez mirror. The prepared electrochemical cell was mounted atop a Pt–Ir electric furnace in the sample holder for operando XAS measurements. The atmospheric *P*(O_2_) around the electrochemical cell was controlled by flowing a mixture of O_2_ and He gases, while ensuring the sufficient penetration of incident and fluorescence X-rays to and from the sample, respectively. *P*(O_2_) in ambient atmosphere was controlled in the range of 1–10^–3^ bar. The temperature was kept constant at 873 K. All electrochemical measurements were performed with a three-terminal configuration. Before operando XAS measurements, AC EIS measurements was carried out under an open circuit with 10 mV of amplitude in the frequency range between 100 kHz and 100 mHz to confirm the proper operation of the cell and to evaluate the ohmic resistance of the electrolyte. During operando micro XAS measurements, the electrode was cathodically polarized by applying a constant DC bias with a potentiostat/galvanostat (VersaSTAT3, AMETEK, Inc.). The voltage and current responses during the polarization are presented in Figure S3. The cathodic overpotential was calculated by subtracting the *IR* drop from the applied cathodic bias. The *IR* drop was estimated from the ohmic resistance of the electrolyte by EIS measurements and from the current observed during DC polarization.

## Supplementary Material



## Notes

Additional details and notes on synthesis and phase identification of materials and polarization behavior during the operando XAS measurements (PDF)

## ∇

Institute of Materials and Systems for Sustainability, Nagoya University, Furo-cho, Chikusa, Nagoya 464-8601, Japan

## #

Faculty of Materials for Energy, Shimane University, 1060 Nishikawatsu-cho, Matue-shi, Shimane 690-8504, Japan.
