# Supplementary material for: Operando Evaluation of the Electrochemically Active Area in a Solid Oxide Fuel Cell Porous Electrode by Micro X‑ray Absorption Spectroscopy
Source: J Phys Chem Lett. 2025 Sep 6;16(37):9599–605. doi: 10.1021/acs.jpclett.5c02422 (PMC12451737; doi:10.1021/acs.jpclett.5c02422)
Supplement: Supplementary file 1 [file jz5c02422_si_001.pdf]

Supporting Information

Operando Evaluation of Electrochemically Active Area in an Solid Oxide Fuel Cell Porous Electrode by Micro X-Ray Absorption Spectroscopy

*Yoshinobu Fujimaki, Takashi Nakamura, Yuta Kimura, Kiyofumi Nitta, Oki Sekizawa, Yasuko Terada, Keiji Yashiro, Tatsuya Kawada and Koji Amezawa**

Material synthesis

Electrolyte: A $Ce_{0.9}Gd_{0.1}O_{1.95}$ powder was synthesized by co-precipitation method of cerium and gadolinium oxalates from a mixed nitrate solution prepared from CeO_2 (Rare Metallic Co., Ltd., 99.99%) and Gd_2O_3 (Furuuchi Chemical Corporation, 99.99%). The obtained powder was calcined at 1073 K for 5 hours in air, and pressed into a pellet by isostatic pressing at 200 MPa. The pellet was sintered at 1823 K for 5 hours. Mirror polishing was performed on one surface of the pellet. The thickness of the $Ce_{0.9}Gd_{0.1}O_{1.95}$ pellet was approximately 2 mm.

Working electrode: A $La_{0.6}Sr_{0.4}CoO_{3-\delta}$ powder was produced by Pechini method. A mixed aqueous nitrate solution was prepared from La_2O_3 (Rare Metallic Co., Ltd., 99.99%), $SrCO_3$ (Rare Metallic Co., Ltd., 99.99%) and $Co(NO_3)_2 \cdot 6H_2O$ (Kojundo Chemical Laboratory Co., Ltd., 99.9%). The obtained solution with excess amount of ethylene glycol and citric acid was heated to 873 K. The remained powder of polymeric precursor was calcined at 1273 K for 5 hours, and $La_{0.6}Sr_{0.4}CoO_{3-\delta}$ powder was obtained.

Phase identification of the materials

The produced powders were analyzed by X-ray diffractometer (Mac Science M18X). Figures S1 and S2 show the 2θ-scan of the synthesized materials. The diffractograms confirm that the powders of $Ce_{0.9}Gd_{0.1}O_{1.95}$ and $La_{0.6}Sr_{0.4}CoO_{3-\delta}$ were a single-phase fluorite and perovskite, respectively. Slight shifts in XRD peaks of prepared $La_{0.6}Sr_{0.4}CoO_{3-\delta}$ compared with those of the reference were caused by the partial reduction of the oxide, *i.e.* the slight increase in oxygen nonstochiometry, δ, due to the high temperature synthesis.

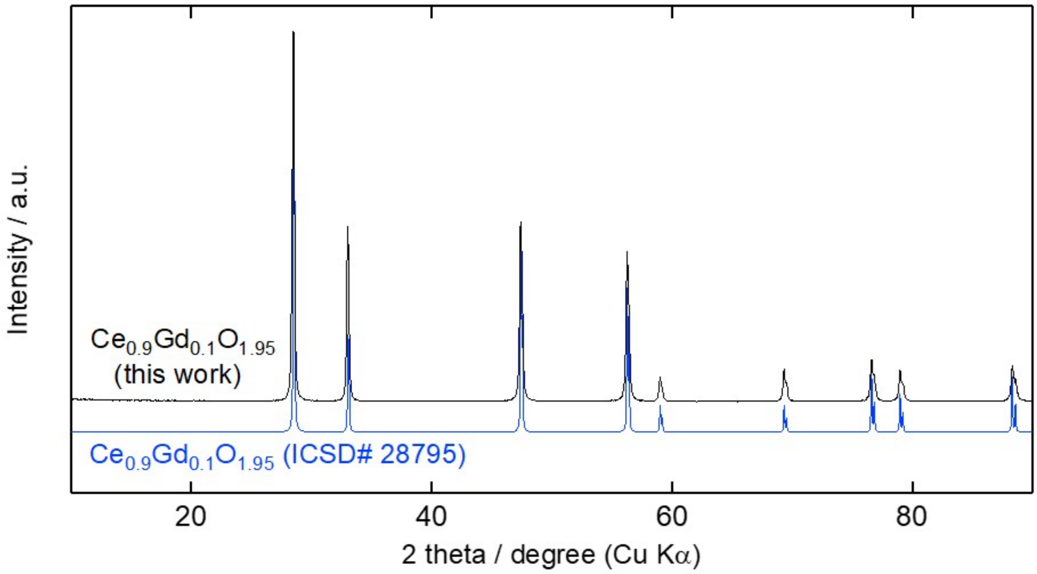

Figure S1. X-ray diffraction patterns of synthesized $Ce_{0.9}Gd_{0.1}O_{1.95}$ powder.

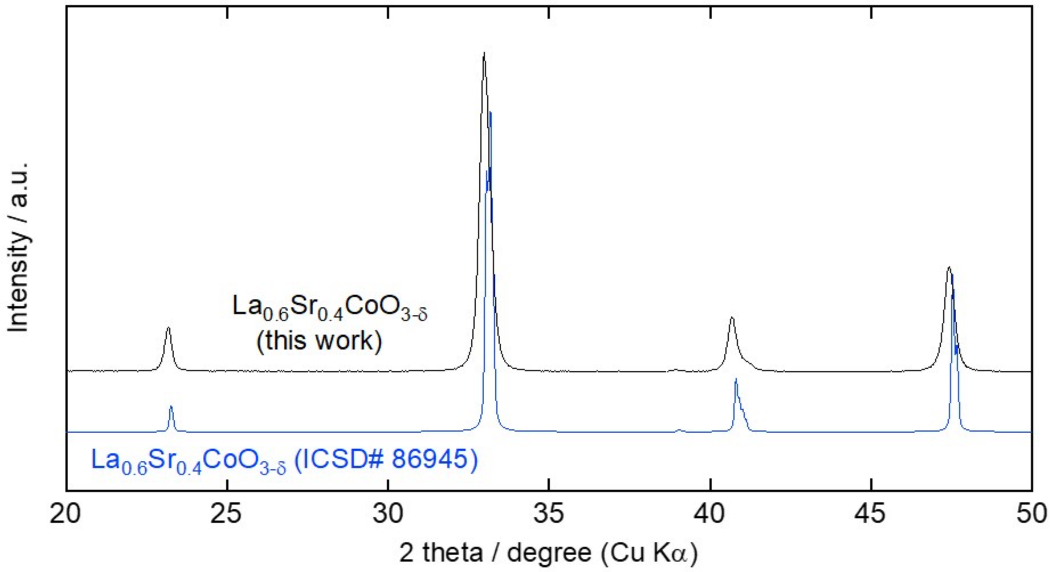

Figure S2. X-ray diffraction patterns of synthesized $La_{0.6}Sr_{0.4}CoO_{3-\delta}$ powder.

Polarization during the operando XAS measurements

During the operando XAS measurements, the $La_{0.6}Sr_{0.4}CoO_{3-\delta}$ was cathodically polarized by applying a constant DC voltage of 700 mV at 873 K under 10^{-2} bar of $P(O_2)$. Figure S3 shows the transient curves of the applied voltage and the observed current. The current was stable at around 2.45 mA during the measurements. By subtracting the ohmic loss, which was calculated

by multiplying the observed current with the ohmic resistance, from the applied voltage, the cathodic overvoltage was evaluated as 140 mV. The ohmic resistance was determined from the AC impedance spectrum in Fig. 4(a) in the main text.

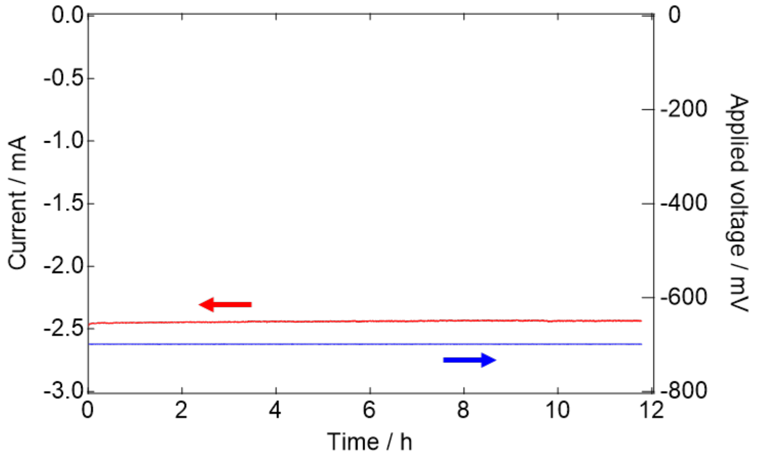

Figure S3. Transient curves of the applied voltage and the observed current during the operando XAS measurements.
